# HMGB2 orchestrates mitotic clonal expansion by binding to the promoter of C/EBPβ to facilitate adipogenesis

**DOI:** 10.1038/s41419-021-03959-3

**Published:** 2021-07-02

**Authors:** Keren Chen, Junyan Zhang, Feng Liang, Qi Zhu, Shufang Cai, Xian Tong, Zuyong He, Xiaohong Liu, Yaosheng Chen, Delin Mo

**Affiliations:** State Key Laboratory of Biocontrol, School of Life Sciences, Sun Yat-sen University, Guangzhou, Guangdong China

**Keywords:** Cell proliferation, Differentiation

## Abstract

High-mobility group box 2 (HMGB2) is an abundant, chromatin-associated protein that plays an essential role in the regulation of transcription, cell proliferation, differentiation, and tumorigenesis. However, the underlying mechanism of HMGB2 in adipogenesis remains poorly known. Here, we provide evidence that HMGB2 deficiency in preadipocytes impedes adipogenesis, while overexpression of HMGB2 increases the potential for adipogenic differentiation. Besides, depletion of HMGB2 in vivo caused the decrease in body weight, white adipose tissue (WAT) mass, and adipocyte size. Consistently, the stromal vascular fraction (SVF) of adipose tissue derived from *hmgb2*^−/−^ mice presented impaired adipogenesis. When *hmgb2*^−/−^ mice were fed with high-fat diet (HFD), the body size, and WAT mass were increased, but at a lower rate. Mechanistically, HMGB2 mediates adipogenesis via enhancing expression of C/EBPβ by binding to its promoter at “GGGTCTCAC” specifically during mitotic clonal expansion (MCE) stage, and exogenous expression of C/EBPβ can rescue adipogenic abilities of preadipocytes in response to HMGB2 inhibition. In general, our findings provide a novel mechanism of HMGB2-C/EBPβ axis in adipogenesis and a potential therapeutic target for obesity.

## Introduction

White adipose tissue (WAT) involves in caloric storage and consumption and plays a considerable role in regulating energy balance and glucose homeostasis [1, 2]. Obesity is tightly associated with the extravagant accumulation of WAT, which mainly results from energy intake in the excess of its consuming [3, 4]. Morbid obesity leads to severe metabolic disease, such as type 2 diabetes, cardiovascular disease, and related cancers [5, 6]. The ongoing metabolic disease has been brought to the forefront of global attention [7, 8]. The primary contributors to the occurrence of obesity are referred as hypertrophy (adipocyte size increase) and hyperplasia (adipocyte number increase) [9]. Adipogenesis was classified into three phases, including cell commitment, early differentiation, and terminal differentiation [10, 11]. After being stimulated by adipogenic inducers, the growth-arrested 3T3-L1 preadipocytes re-enter a specific cell-cycle period, termed as mitotic clonal expansion (MCE) [12, 13]. In this stage, the number of cells increases about fourfold and terminal differentiation is activated [14, 15]. Multiple adipogenic genes are highly expressed and transcriptional factor cascades are activated during MCE [11, 14]. Disruption of MCE will prevent the events of adipocytes differentiation [16, 17]. Therefore, MCE is an obligatory stage for preadipocytes differentiating into adipocytes [18, 19].

CCAAT/enhancer-binding protein-β (C/EBPβ), a member of leucine zipper family, has been confirmed to play a vital role in regulating adipogenic cascades during early adipogenesis [20]. Normally, C/EBPβ is activated promptly after confluence 3T3-L1 induced by hormonal inducers, followed by transactivating the expression of peroxisome proliferator-activated receptor-γ (PPARγ) and C/EBPα [21, 22]. Then, they both motivate lipogenesis-related factors that are necessary for lipid formation and accumulation, such as perilipin and SREBP 1c [10, 23]. Deficiency of C/EBPβ in vitro inhibits adipogenesis, while its overexpression promotes adipogenesis, even without inducers [24, 25]. Further, disruption of C/EBPβ expression in vivo causes reduced fat mass due to the impairment of adipose tissue development [26]. Thus, C/EBPβ is an essential early adipogenic factor to promote adipogenesis [27].

High-mobility group box 2 (HMGB2) belongs to the protein family of HMGB and has been confirmed to participate in multiple cellular events, such as DNA replication, DNA repair, and gene transcription [28, 29]. Previous research has reported that the loss of HMGB2 in cells leads to the changes of transcription [30]. Besides, HMGB2 regulates muscle regeneration and enhance human topoisomerase in tumors [31, 32]. It highly expresses in undifferentiated mesenchymal stem cells (MSC) and coordinates with platelet-derived growth factor receptor-α (PDGFRα) to regulate adipocytes differentiation through early adipogenic signal cascade [33]. In our previous study, it was found that HMGB2 expressed higher in both subcutaneous and intramuscular stromal vascular cells [34]. After adipogenic induction, it decreased remarkably. However, the molecular mechanism regarding the role of HMGB2 in adipogenesis is not well understood.

Here, we found HMGB2 ablation diminishes adipogenic abilities in vivo. Moreover, its deficiency inhibits cell MCE in vitro. Our data provide solid evidence that HMGB2 promote adipogenesis via binding to the site “GGGTCTCAC” of C/EBPβ promoter in MCE stage.

## Results

### HMGB2 is required for preadipocyte differentiation

**Fig. 1:**
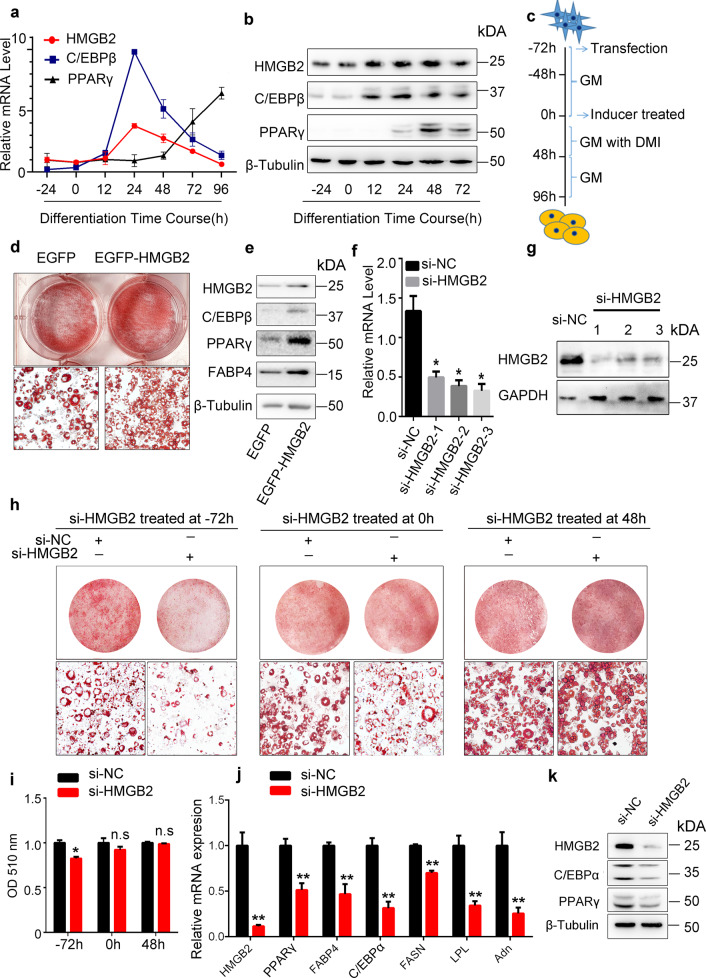
HMGB2 is required for adipogenesis. **a** qPCR for mRNA of HMGB2, C/EBPβ, and PPARγ during 3T3-L1 cells differentiation. **b** Western blot analysis of protein levels of HMGB2, C/EBPβ, and PPARγ. **c** Schema of si-HMGB2 or EGFP-HMGB2 plasmid treatment during adipogenic induction. **d** Oil Red-O staining of cells treated with EGFP-HMGB2 plasmid. **e** Western blot analysis of EGFP-HMGB2 plasmid in 3T3-L1 cells. **f**, **g** Knockdown efficiency of three si-HMGB2 examined by qPCR and western bot. **h** Effect of siRNA transfection at different time points on adipogenesis, as determined by Oil Red-O staining. **i** Statistical data of Oil Red-O by OD measurements. **j** qPCR for mRNA of HMGB2 and adipogenic genes. **k** Western blot analysis for HMGB2, C/EBPα, and PPARγ. Data represent three biological replicates each with three technical replicates. (**f**, **j**: *n* = 3; data presented in graphs represent mean s.e.m. *P < 0.05; **P < 0.01, Students’ *t* test. **d**, **e**, **h**: cells were harvested and stained at day 10, *n* = 3).

To understand the role of HMGB2 in adipogenesis, the endogenous expression profiles of HMGB2, C/EBPβ, as well as PPARγ in vitro were examined. The result showed that the mRNA levels of HMGB2 and C/EBPβ promptly increased after DMI induction and reached the peak at 24 h, then gradually reduced, whereas PPARγ expressed at late stage of adipogenesis (Fig. 1a, b). The result indicated that the expression trend of HMGB2 was similar to C/EBPβ, but not PPARγ. Following the schema of HMGB2 overexpression (Fig. 1c), 3T3-L1 cells exhibited enhanced adipogenic abilities with increasing triglycerides formation, lipid accumulation, and upregulation expression of adipogenic factors, including C/EBPβ, PPARγ, and FABP4, as confirmed by Oil Red-O staining (Fig. 1d) and western blot (Fig. 1e), respectively. Conversely, three individual Oil Red-O staining experiments were conducted in 3T3-L1 cells, and the results revealed that HMGB2 knockdown at −72 h before DMI induction was able to significantly inhibit adipocyte differentiation and lipid accumulation, while HMGB2 interference at 0 or 48 h after DMI showed no effects on adipogenesis (Fig. 1f, g, h), as confirmed by optical density (OD) measurements (Fig. 1i). Further, knockdown of HMGB2 at −72 h inhibited the expression of terminal adipogenic genes, including PPARγ, C/EBPα, FABP4, etc. (Fig. 1j, k). These results demonstrated that HMGB2 influences adipogenesis exclusively at the early differentiation stage. Together, these results indicate that HMGB2 is necessary for adipogenesis.

### HMGB2 exclusively involved in MCE in adipogenesis

**Fig. 2:**
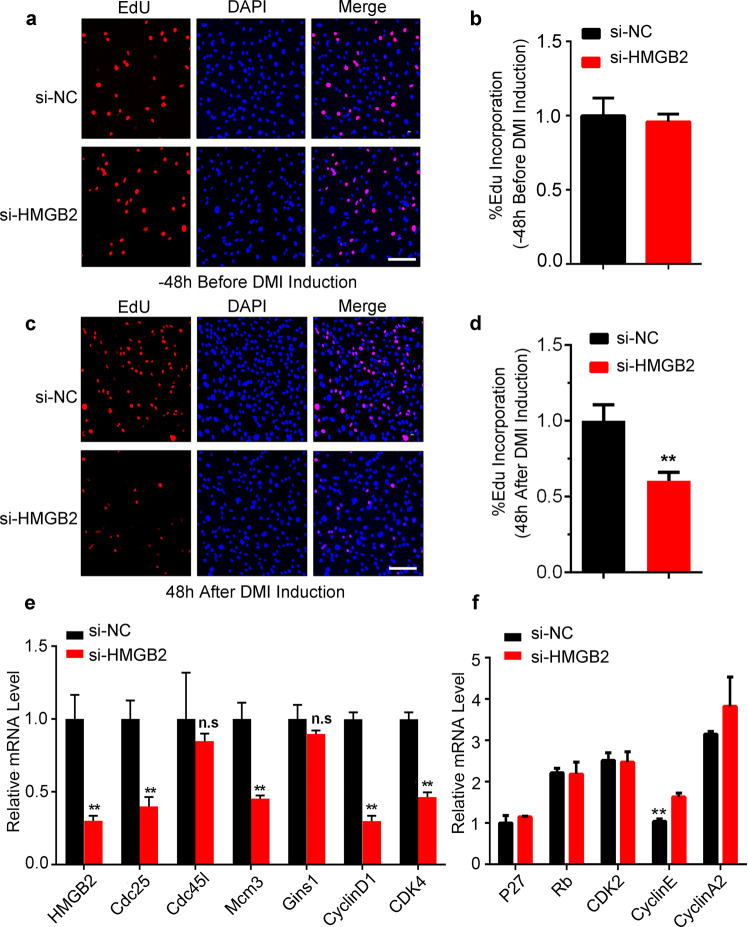
HMGB2 promotes mitotic clonal expansion in adipogenesis. **a**–**d** Detection of cell proliferation at different stages after HMGB2 interference by using immunofluorescence assay. **e, f** qPCR for mRNA of MCE-specific genes and cell-cycle genes affected by si-HMGB2 in 3T3-L1 cells. (**b, d**–**f**: *n* = 3, **P < 0.01; *P < 0.05 against si-NC, Students’ *t* test; data presented in graphs represent mean ± s.e.m.).

Although the above-mentioned results have indicated that HMGB2 influences adipogenesis at early differentiation phase, the mechanism about how HMGB2 regulates early adipogenesis remains unclear. To address this, 5‐ethynyl‐2′‐deoxyuridine (EdU) incorporation assay was carried out at different period to check the interference effects resulted from knocking down HMGB2 on cell proliferation at different stages. As a result, no change occurred for the percentage of EdU-positive cells detected at −48 h (normal cell proliferation stage) (Fig. 2a, b). However, when detected at MCE stage (48 h after DMI induction), the percentage of EdU-positive cells significantly reduced by si-HMGB2 treatment at same stage (−72 h) (Fig. 2c, d). Besides, MCE-specific genes, including Mcm3, Gins1, CyclinD1, and CDK4, were predictably attenuated after HMGB2 knockdown (Fig. 2e). However, cell-cycle genes expressed at normal cell proliferation (detected at −48 h) were not affected by HMGB2 knockdown (Fig. 2f). These results suggest that HMGB2 specifically involves in regulating MCE.

### Deletion of HMGB2 suppresses adipogenesis in vivo

**Fig. 3:**
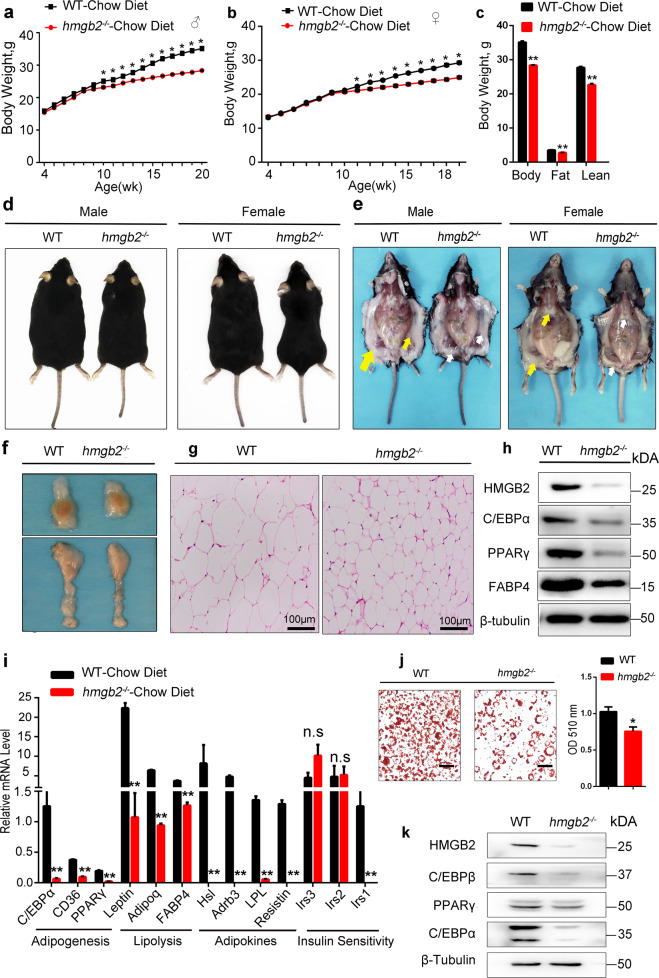
Deletion of HMGB2 suppresses adipogenesis in vivo. **a**, **b** Representative body weight diagram of male (*n* = 5) and female mice (*n* = 6). **c** The weight of Body, fat tissue, and lean of *hmgb2*^−/−^ and WT mice. *n* = 5, male, 12 weeks old. **d** The phenotype of WT and *hmgb2*^−/−^ mice at 12 weeks postnatal. **e** Dissection images. Yellow arrow: ingWAT in WT mice; white arrow: ingWAT in *hmgb2*^−/−^ mice. **f** Anatomical images of eWAT (upper picture) and ingWAT (lower picture). **g** H&E staining of ingWAT slides. Scale bar: 100 μm. **h** Western blot analysis of protein level for adipogenic maker genes. Protein was extracted from ingWAT of male mice (*n* = 5). **i** qPCR analysis for genes related to adipogenesis, lipolysis, adipokines, and insulin in ingWAT. **j** Oil Red-O staining of SVFs derived from ingWAT (left) and OD measurements of stained cells (right). **k** Western blot analysis of protein levels for adipogenic marker genes in SVFs. (**i**: mean values ± s.e.m. **P < 0.01; *P < 0.05).

Technically, *hmgb2*^−/−^ mice were generated by CRISPR-cas9 (Supplementary Fig. 1a, b). Compared with wild type (WT), *hmgb2*^−/−^ mice had no significant alteration in body weight from birth to 10 weeks when fed with chow diet. However, 10 weeks later, *hmgb2*^−/−^ mice had a lower body weight, both in male and female (Fig. 3a, b). To compare the differences of phenotypic traits related to fat deposition between WT and *hmgb2*^−/−^ mice, the fat and muscle tissues were completely isolated from the mice and weighed at 12 weeks. Predictably, we found that the fat and lean weight decreased significantly in *hmgb2*^−/−^ mice (Fig. 3c). At the same time, *hmgb2*^−/−^ mice showed a smaller body size (Fig. 3d), accompanied by smaller adipocyte tissue depots (Fig. 3e). Moreover, representative images of inguinal and epididymal WAT (eWAT) depot sections indicated that HMGB2 deletion inhibited fat deposition (Fig. 3f). H&E staining results demonstrated that the size of adipocytes derived from WAT of *hmgb2*^−/−^ mice was remarkably smaller (Fig. 3g). Consistent with this, HMGB2 ablation led to reduced expression of PPARγ, C/EBPα, and FABP4, which are supposed to highly expressed in terminal adipocyte differentiation (Fig. 3h). Quantitative real-time PCR (qPCR) assay also manifested that adipogenesis-involved genes, lipolysis-related factors, adipokine-associated genes, and some insulin (INS) sensitivity-relevant genes were downregulated after HMGB2 deletion, except Irs2 and Irs3 (Fig. 3i). In addition, the adipogenic ability of stromal vascular fractions (SVFs) derived from inguinal WAT (ingWAT) of *hmgb2*^−/−^ mice was also attenuated (Fig. 3j). The expression level of adipogenic genes, including PPARγ, C/EBPα, and C/EBPβ, were reduced, as confirmed by western blot (Fig. 3k). Thus, the manipulated deletion of HMGB2 in vivo led to decrease in body weight, smaller fat depot size, and weakened adipogenic potential in mice, along with the reduced expression of adipogenic genes.

### High-fat diet (HFD) enhances adipogenic ability of *hmgb2*^−/−^ mice but at a lower rate

**Fig. 4:**
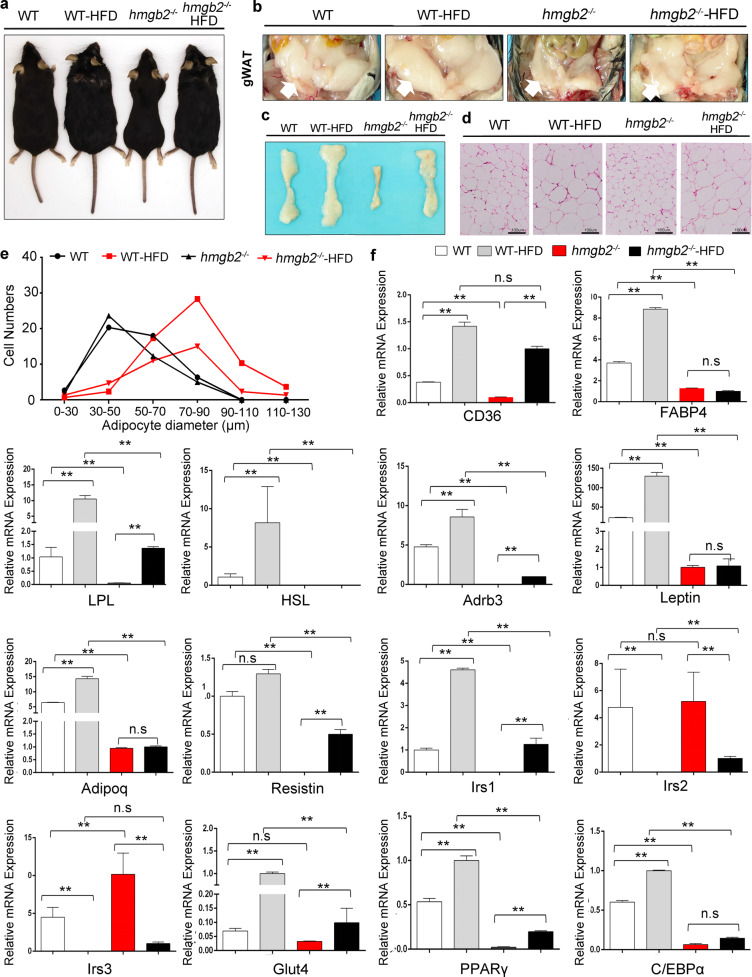
HFD enhances adipogenic ability of *hmgb2*^−/−^ mice but at a lower rate. **a** Representative comparison images of 4 different groups. **b**, **c** Abdominal WAT and ingWAT images of four mice groups. **d** H&E staining of ingWAT slides. **e** Statistical data of adipocyte size measurements from four groups. **f** qPCR of adipogenic genes of four mice groups. (four groups of mice: WT (chow diet), *hmgb2*^−/−^ (chow diet), WT HFD, and *hmgb2*^−/−-^HFD. **a**–**d**: male; age: 20 weeks old; *n* = 5, **f**: mean values ± s.e.m. **P < 0.01).

To evaluate how *hmgb2*^−/−^ mice reacted to overnutritional condition, HFD feeding was carried out. As a result, *hmgb2*^−/−^ mice with HFD showed increased body weight, fat and lean weight as well as body size, but smaller than WT mice fed with HFD (Fig. 4a and Supplementary Fig. 2a–d). The histological results indicated that the smallest abdominal WAT and ingWAT were found in *hmgb2*^−/−^ mice (Fig. 4b and Supplementary Fig. 2e). When fed with HFD, the fat pad of *hmgb2*^−/−^ mice was increased, but smaller than that of WT mice fed with HFD (Fig. 4b, c). In addition, fatty liver only occurred in WT mice fed with HFD rather than other mice groups, which means HMGB2 knockout in vivo caused impaired lipid formation in liver, even fed with HFD (Supplementary Fig. 2f). Expectedly, H&E staining on ingWAT and abdominal WAT revealed that the adipocytes size of *hmgb2*^−/−^ mice fed with HFD was significantly larger than that of *hmgb2*^−/−^ mice fed with chow diet, even bigger than that of WT mice nurtured with chow diet (Fig. 4d and Supplementary Fig. 2g), which was further confirmed by statistical data about the diameters of adipocytes (Fig. 4e and Supplementary Fig. 2h). Consistently, the expression of adipogenic genes in *hmgb2*^−/−^ mice was promoted remarkably by HFD feeding, such as PPARγ, C/EBPα, LPL, lrsl, and Glut4, but still less than that in WT mice fed with HFD (Fig. 4f). Together, HFD promotes adipogenesis in *hmgb2*^−/−^ mice, but not reaching the level of WT mice.

### Screening of HMGB2-target genes at MCE period

**Fig. 5:**
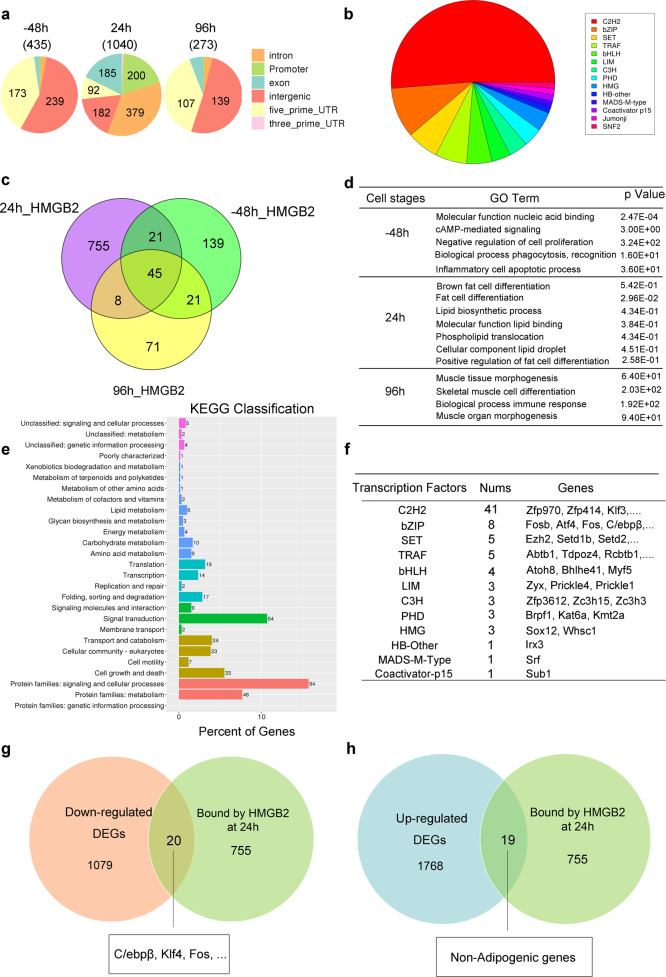
Analysis of HMGB2 binding targets at different stages of adipogenesis. **a** Genomic distribution analysis of HMGB2 binding regions using ChIP-seq data at different sampling time points. **b** Classification analysis of HMGB2-target genes at MCE stage by iTAK software (version 1.7a). **c** Venn diagram for the numbers of genes bound by HMGB2 on different sampling time points. **d** GO enrichment for the biological process that HMGB2 participates in. **e** KEGG classification of HMGB2 at 24 h. **f** Classification of transcription factors and the numbers that bound by HMGB2. **g, h** Venn diagram about genes screened using RNA-seq and ChIP-seq data at 24 h.

To screen the target genes bound by HMGB2, chromatin immunoprecipitation (ChIP) experiments were performed on 3T3-L1 cells harvested at −48 h (proliferation stage), 24 h (MCE stage), and 96 h (differentiation stage) relative to DMI induction (0 h), respectively. As a result, the distribution of HMGB2 binding sites showed significant difference at MCE phase in contrast to cell proliferation and terminal differentiation stages (Fig. 5a and Supplementary Fig. 3a–c). Further, there are about three times more binding sites of HMGB2 in MCE stage than that in others, and these sites mainly distributed on the promoters, exons, and introns, which are the main binding region of transcription factors. Classification of HMGB2 binding peaks on its targets showed that HMGB2 highly binds to the factors of C2H2, bZIP, bHLH, HMG family, and so on (Fig. 5b). Among these factors, C/EBPβ belongs to bZIP transcription factor family. According to the Venn diagram of HMGB2-target genes at three different phases, the number of target genes in MCE stage were much more than −48 and 96 h, in which 755 genes were found exclusively in MCE stage (24 h) (Fig. 5c). This result suggested that HMGB2 functions more actively during MCE phrase. Moreover, only in MCE stage, the targets of HMGB2 mainly participated in fat cell differentiation and lipid biosynthetic process, while at −48 or 96 h, the targets involved in nonadipogenic biological process (Fig. 5d). Based on the 755 target genes, KEGG classification in 24 h indicated that these targets participate in lipid metabolism, energy metabolism, and other important biological processes (Fig. 5e and Supplementary Fig. 3d). From the result of ChIP-seq, a series of HMGB2-target genes in 24 h including ATF4, Fos, C/EBPβ, etc. were found (Fig. 5f). Combining downregulated DGEs and ChIP-seq data, a total of 20 genes that bound by HMGB2 were obtained only at 24 h, including C/EBPβ, Fos, KLF4, etc. Among the upregulated genes, there were 19 genes bound by HMGB2 only at 24 h, but they were not involved in adipogenesis (Fig. 5g, h and Supplementary Fig. 4a, b). Furthermore, transcriptome analysis was performed on ingWAT derived from *hmgb2*^−/−^ mice and WT mice, and the results indicated that the key genes closely related to adipogenesis, such as C/EBPβ, C/EBPδ, PPARγ, KLF4, and C/EBPα, were all downregulated in *hmgb2*^−/−^ mice (Supplementary Fig. 4a–f). To sum up, HMGB2 functions principally in MCE stage to modulate adipogenesis.

### HMGB2 binds to C/EBPβ promoter to facilitate adipogenesis

**Fig. 6:**
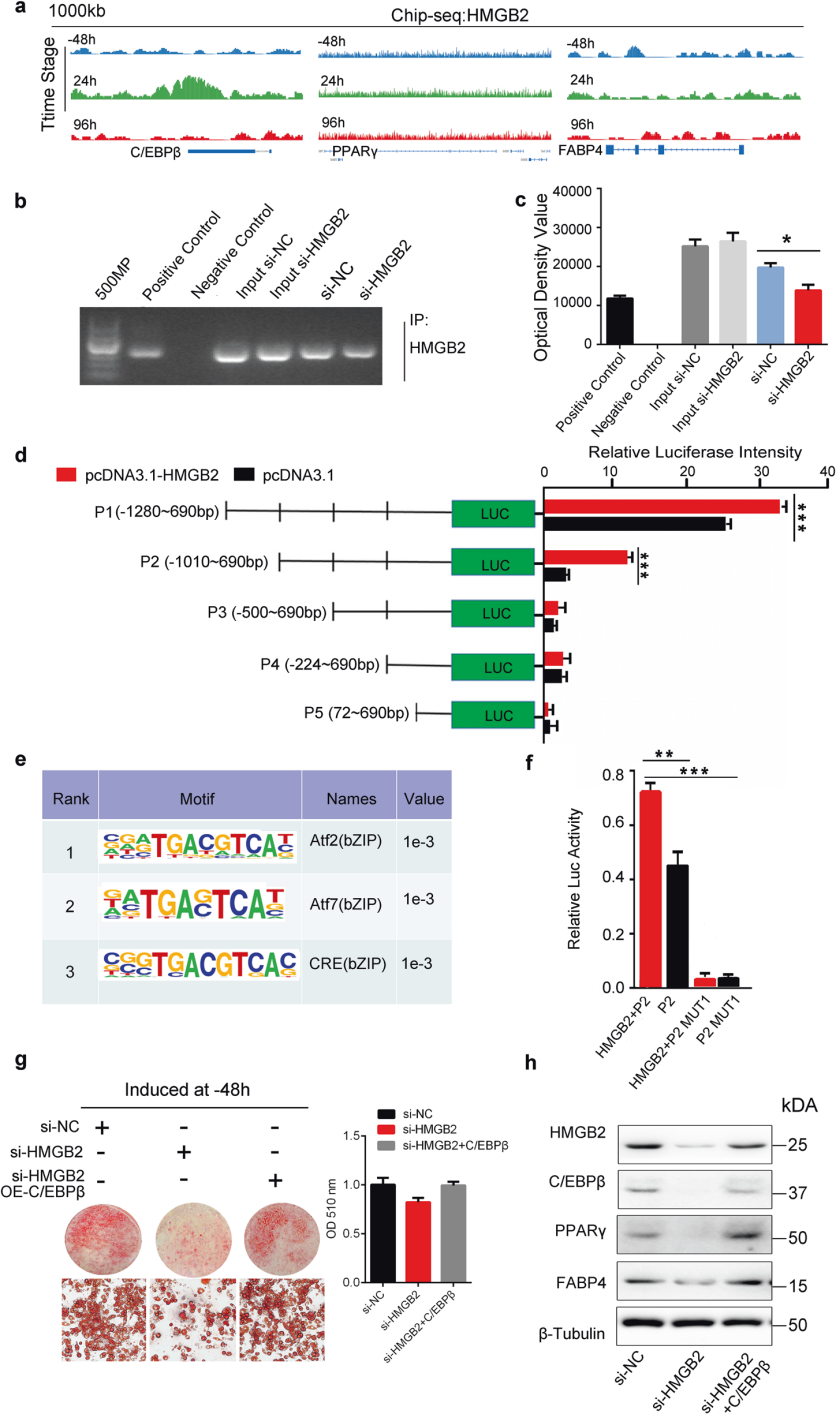
HMGB2 binds to the promoter of C/EBPβ in MCE stage to modulate adipogenesis. **a** ChIP-seq analysis of the peak distributions of C/EBPβ, PPARγ, and FABP4 loci bound by HMGB2 at −48, 24, and 96 h. **b** ChIP-PCR assay for the interaction between HMGB2 and C/EBPβ promoter in 3T3-L1 cells. **c** Band intensity for **b** (*n* = 3, *P < 0.05). **d** Luciferase activity for truncated DNA fragments of C/EBPβ (***P < 0.001). Experiments were conducted in 293T cells. **e** Binding motif of HMGB2 on C/EBPβ. HOMER and KNOWN analysis by ChIP-seq on FDR-corrected, *p* values represent enrichment. **f** Luciferase activity of potential regions of C/EBPβ bound by HMGB2 before and after mutation (***P < 0.001). Experiments were carried out in 293T cells. **g** Oil Red-O staining assay in 3T3-L1 cells (left) and OD measurements (right). **h** Western blot for adipogenic genes. (**g**, **h**: 3T3-L1 cells transfected with si-NC, si-HMGB2, and si-HMGB2 with C/EBPβ overexpression plasmid, respectively).

Considering the importance of C/EBPβ in the MCE stage, it was selected as the candidate gene bound by HMGB2 in this study. ChIP-seq data showed that peak of C/EBPβ enriched by HMGB2 only in MCE stage (24 h) rather than other stages, which was confirmed by the chromatin peak landscape of HMGB2 binding to the promoters of C/EBPβ at −48, 24, and 96 h relative to DMI induction, respectively (Fig. 6a and Supplementary Fig. 5). Meanwhile, there were no significant enrichment changes of HMGB2 on PPARγ and FABP4 at −48 or 96 h. This result indicated that HMGB2 only bound to the promoter of C/EBPβ during the MCE period, and would not bind to the key genes for terminal differentiation and lipolysis. Further, when the expression of HMGB2 was knocked down, the enrichment of HMGB2 on C/EBPβ promoter was significantly decreased, which was confirmed by ChIP-PCR (Fig. 6b, c).

To screen HMGB2 binding sites that can enhance C/EBPβ transcription, a series of truncated DNA fragments from a 2 kb region around the transcription start site (TSS) of C/EBPβ were generated and inserted into pGL3-Basic vector (Supplementary Fig. 6a). After transfection into 293T cells, the fluorescence intensity was measured and showed that the binding region with the highest transcriptional activity located at fragment P1 (−1280–690 bp, relative to TSS) of the C/EBPβ promoter, followed by P2 (−1010–690 bp), whereas the luciferase intensity of P3 (−500–690 bp), P4 (−224–690 bp), and P5 (72~690 bp) was almost undetectable (Fig. 6d). These results indicated that the effective promoter regions of C/EBPβ bound by HMGB2 was mainly located in P1 and P2, and the latter harbors three predicted binding motifs related to bZIP family from different region between P2 and P3 (Fig. 6e). When the site “GGGTCTCAC” located at −889 bp of C/EBPβ was mutated, the transcriptional activity almost completely lost even overexpressing HMGB2 (Fig. 6f and Supplementary Fig. 6b, c), which indicated that this site is indispensable to promote transcription of C/EBPβ by recruiting HMGB2. In addition, it showed that the attenuated lipid accumulation caused by si-HMGB2 was rescued by overexpressing C/EBPβ (Fig. 6g and Supplementary Fig. 7a, b), which was confirmed by the protein level of adipogenic genes and Oil Red-O staining (Fig. 6h and Supplementary Fig. 7c).

## Discussion

HMGB2 belongs to HMG family that are architectural factors regulating DNA repair, recombination, replication, and transcription [35, 36]. Previous research has confirmed HMGB2 ubiquitously distributes in nucleus of eukaryotes and involves in multiple biological processes [28]. Functionally, HMGB2 can bend DNA and form DNA circles to participate in biological activities [37]. Previous researches have shown that myoblast, chondroblast, osteoblast, and adipocytes are originated from same precursors before commitment [4, 38]. As a chromatin binding protein, HMGB2 was reported to be involved in regulating senescence-associated secretory phenotype [39]. Besides, HMGB2 has been proven to play an important role in mediating myogenesis due to interacting with IGF2BP2 to control skeletal muscle regeneration [31]. In addition, HMGB2 can regulate chondrogenesis of MSC [35]. The enhanced expression of HMGB2 promotes adipogenesis of MSCs and infiltration of fat into skeletal muscle through PDGFRα [33]. Moreover, it was reported that HMGB2 expressed highly before DMI induction and expressed lower after differentiation in porcine intramuscular fat cells, whereas its expression trend was entirely different from the expression profile of PPARγ and FABP4 [34]. More than that, HMGA2, the homologous protein of HMGB2, also enhances adipogenesis via initiating PPARγ expression [40]. Although the role of HMGB2 in adipogenesis has been reported, the regulatory mechanism of HMGB2 promoting adipogenesis is still unclear. In our study, it was confirmed that HMGB2 orchestrates adipogenesis by binding to the promoter of C/EBPβ only in MCE stage.

During the earlier adipogenic phase, MCE is considered as the cross-talk between cell proliferation and differentiation in adipogenesis [19, 24]. In this study, deficiency of HMGB2 impairs MCE of 3T3-L1 cells rather than normal cell proliferation before DMI induction. This result is consistent with that using dominant-negative C/EBPβ in 3T3-L1 cells inhibits MCE specifically [24]. It was also reported that impairment of MCE leads to inhibition of adipocyte differentiation [16, 41]. To explain this phenomenon, a report indicated that when preadipocytes go through checkpoint of G(1)S, C/EBPβ needs activity of DNA-binding to activate a series of adipogenic transcription factors [15]. Interestingly, HMGB2 is a DNA-binding protein to participate in biological processes [42, 43], which leads us to think it is HMGB2 that provided DNA-binding activity with C/EBPβ in MCE stage to initiate downstream adipogenic transcription activities. Certainly, due to the importance of MCE, there are many other genes participated in this process. For instance, KDM4b was confirmed to cowork with C/EBPβ to mediate adipogenesis at MCE [44]. FTO was identified to affect MCE to influence adipogenesis as well [18].

Most of previous research considered C/EBPβ as the transcription factor which binds to enclosed chromatin [45, 46], and plays a pioneer role to activate tissue-specific transcription activities at genes’ promoters [47]. However, few studies have focused on C/EBPβ as a target gene bound by other transcription factor in adipocyte differentiation, except KLF4 [48] and Krox20 [49]. In our study, multiple evidences have demonstrated HMGB2 binds to the promoter of C/EBPβ to promote adipogenesis.

It was showed that HMGB2 knockout mice exhibited severe osteoarthritis [50], and their fertility decreased, which was due to Sertoli and degeneration of germ cell in immotile spermatozoa and seminiferous tubules [51]. However, the adipogenic phenotype of *hmgb2*^−/−^ mice has not been described before. In our study, it indicated that *hmgb2*^−/−^ mice showed impaired adipogenesis, which is mainly due to the smaller size of adipocytes. Moreover, HFD could enhance body size and fat pads, but the *hmgb2*^−/−^ mice were still smaller than WT mice fed with HFD. Some research confirmed that HFD mainly promote the rapid accumulation of energy in animals to achieve rapid adipocyte hypertrophy [52, 53]. Our results present that HFD restores the size of WAT in *hmgb2*^−/−^ mice, but it has no compensation for the regulatory effect of HMGB2. These results further indicated that HMGB2 is involved in modulating a cascade of early adipogenesis rather than lipid formation.

**Fig. 7:**
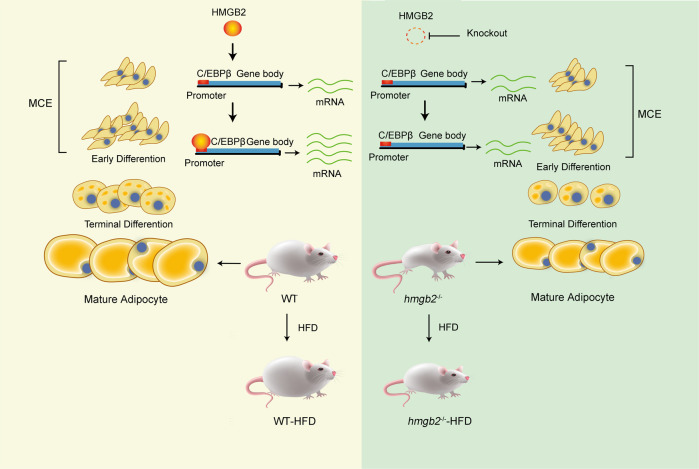
Regulatory mechanisms of HMGB2 in adipogenesis. HMGB2 binds to the promoter region of C/EBPβ in MCE stage to regulate adipogenesis. High-fat diet enhances adipogenic ability of *hmgb2*^−/−^ mice but at a lower rate.

In summary, HMGB2 plays an important role in regulating adipogenesis by binding to the promoter of C/EBPβ in MCE stage. When knocking down HMGB2, the interaction between HMGB2 and C/EBPβ was weakened as confirmed by ChIP-PCR. Due to the loss of HMGB2, the expression of C/EBPβ was attenuated to repress MCE, leading to the inhibition of adipogenesis, smaller mature adipocytes and body size in vivo (Fig. 7).

## Materials and methods

### Animal study

*hmgb2*^−/−^ mice were generated via CRISPR/Cas9 system. Two single guide RNAs (sgRNAs) targeting the exons 2–5 of HMGB2 were designed on both sides of sequences to make this site fully deleted. This design is based on transcript-201 (NM_008252) which is one of four transcript variants. CRISPR/Cas9 components, including ssDNA donor with Cas9 mRNA and the two sgRNAs, were coinjected into mouse zygotes, followed by transplanting into oviducts of pregnant female mice. F0 mice were crossed with C57BL/6J WT mice to obtain F1 offspring with stable genotypes. All mice used in this research were maintained in SPF conditions and the house facility maintained homoiothermal 22 °C. Four weeks after birth, mice littermates were divided into four groups, WT chow-diet mice, *hmgb2*^−/−^ chow-diet mice, WT HFD mice, and *hmgb2*^−/−^ HFD mice, respectively. All HFD contains 60% fat, whereas the chow diet only contains about 11.5% fat, 17.5% protein, and 2.5% fiber, with the remainder consisting of complex carbohydrates. Body weight of mice from each group was measured every 3 days. The whole animal experimental procedures met the requirements and the ethical standards of the Animal Care and Use Committee of Guangdong Province. Permit numbers are SCXK (Guangdong) 2011-0029 and SYXK (Guangdong) 2011-0112. sgRNAs for generating *hmgb2*^−/−^ mice (5′ to 3′) were listed in Supplementary Table 1. Primers for genotyping of *hmgb2*^−/−^ mice (5′ to 3′) were listed in Supplementary Table 2. In this study, 10 male mice and 12 female were used. Among these mice, five male mice and six female mice were fed with chow diet and the rest were fed with HFD. Mice were randomly allocated to chow-diet group or HFD group.

### 3T3-L1 cells culture and differentiation

3T3-L1 cells used in the study were purchased from American Type Culture Collection and cultured in Dulbecco’s modified Eagle’s medium (DMEM) (Sigma) supplemented with 10% fetal bovine serum (FBS) and 1% streptomycin/ penicillin (growth medium, GM) at 37 °C in the humid incubator with 5% CO_2_. To induce differentiation, postconfluent preadipocytes were treated with hormonal inducers DMI [1 μg ml^−1^ INS (Roche Diagnostics GmbH, Mannheim, Germany), 0.5 mM 3-iso-butyl-1-methylxantine (Sigma), and 1 μM dexamethasone (DEX) (Sigma) in DMEM containing 10% FBS]. Three days later, 3T3-L1 cells were cultured in DMEM supplemented with 10% FBS and 1 μg ml^−1^ INS for 2 days. Then, 3T3-L1 cells were cultured in GM without any inducers at the final differentiation stage.

### RNA interference and overexpression

Three Stealth RNAi™ against mouse HMGB2 (si-HMGB2) (Invitrogen, Shanghai, China) were used to perform RNA interference. The information of siRNA sequences is provided in Supplementary Table 3. The negative RNA supplemented with similar GC content medium (Invitrogen) was conducted as control RNA (si-NC). To conduct gene overexpression, CDS regions of mouse *hmgb2* (NCBI Sequence ID: 97165) and *C/EBPβ* (NCBI Sequence ID:12608) were inserted into pcDNA3.1 vector (Invitrogen), respectively. 3T3-L1 preadipocytes cultured in six-well plates were transfected with siRNA or plasmid at 80% confluency with Lipofectamine 3000 (Invitrogen) according to the manufacturer’s instruction. Fresh GM replaced the transfection mixture medium after 6 h treatment. All the transfection experiments were performed in triplicates.

### Isolation of SVF and collection of adipocyte

SVF was obtained from ingWAT. Briefly, mouse WAT was minced and digested with 0.1% collagenase type II (Sigma) in Krebs Ringer phosphate buffer (KREBs) at 37 °C for 45 min with shaking. Cell suspension was then passed through 100 μm mesh and separated by centrifugation at 300 × *g* for 5 min. Floating adipocyte fraction was collected for RNA or DNA extraction, whereas cell pellets were resuspended in KREBS buffer, passed through 40 mm mesh, and cultured in vitro.

### RNA isolation, RT-PCR, and qPCR analysis

Total RNA was isolated using TRIzol (Invitrogen) and cDNA was synthesized by reverse transcribing from 1 μg total RNA following the manual instructions of Reverse Transcription Kit (Promega, Shanghai, China). The RNA concentration was measured with a NanoDrop spectrophotometer (Thermo Scientific). qPCR was conducted on the system of LightCycler 480 (Roche, Basel, Switzerland) through using SYBR Green qPCR mixture (Dongsheng Biotech, Guangzhou, China). GAPDH was used as the internal control to normalize the qPCR data. Primers for qPCR were collected in Supplementary Table 4.

### RNA sequencing and data analysis

For RNA-seq, total RNA was isolated from ingWAT of *hmgb2*^−/−^ and WT mice. The cDNA libraries were sequenced on the Illumina sequencing platform by Gene de novo Biotechnology Co., Ltd (Guangzhou, China). The parameters for screening differentially expressed genes are based on FDR < 0.05 and |log2FC| > 1, fold change > 2.

### Western blot

Protein was obtained by collecting 3T3-L1 cells or WAT in lysis buffer, containing 150 mM NaCl, 50 mM Tris, 1% sodium deoxycholate, 0.1% SDS, and 1% Triton X-100 as well as fresh protease inhibitor PMSF. Protein was run by gel electrophoresis on SDS-PAGE, followed by transferring to PVDF membrane (Roche). Membranes were sealed with 5% BSA/TBST for 2 h, followed by incubation with primary antibodies at 4 °C overnight. The membranes were washed by phosphate buffer saline (PBS) three times and 5 min each time before incubated with secondary antibodies. Protein bands were captured by the GelView 6000 Pro (BLT, Guangzhou, China). Primary antibodies used were shown as following: anti-HMGB2 (EPR6301, Abcam), anti-C/EBPβ (E299, Abcam), anti-C/EBPα antibody (EP709Y, Abcam), anti-PPARγ antibody (EPR18516, Abcam), anti-FABP4 antibody (EPR3579, Abcam), anti-GAPDH antibody (Loading Control, Abcam), and anti-β-Tubulin antibody (EPR16778, Abcam).

### H&E staining

ingWAT and eWAT from WT and *hmgb2*^−/−^ mice were fixed with 4% paraformaldehyde at 4 °C for 20 h, followed by dehydration with gradient ethanol and paraffin embedding. Then, embedded tissues were cut into sections at a thickness of 3 μm, via operating STP120, EC350, and HM340 (MICROM, Germany) according to the manufacturer’s instruction. Then, tissue sections were rehydrated by gradient ethanol and H_2_O, and immersed in hematoxylin for 10 min followed by eosin for 5 min, then dehydrated in alcohol and treated with xylene for decoloration. The images of WAT tissues were captured when they were isolated within 20 min.

### Adipogenic induction and Oil Red-O staining

Growth-arrested 3T3-L1 preadipocytes were induced by DMI inducer containing GM with 1 μM of DEX, 0.5 mM of 1-methyl-3-isobutylxanthine, 5 μg/ml INS. After 3-day DMI induction, cells were cultured in GM with 5 μg/ml INS (INS GM) for another 2 days. Then, the INS GM were replaced with GM for another 3 days. On the sixth day after DMI induction, mature adipocytes were carefully washed with PBS, followed by infiltrated in 4% paraformaldehyde for 30 min–1 h at room temperature. Then, cells were stained in the 60% saturated Oil Red-O reagent (Sigma-Aldrich) for 30 min, followed by washed with 40% ethanol and PBS for three times, 2 min for each time, respectively. Following the above-mentioned methods, cells could be visualized and examined under microscope through Bright field imaging under Nikon TE2000 microscope (Nikon, Tokyo, Japan). The Oil Red-O absorbed in the cells was extracted by 100% isopropanol and examined by Synergy 2 Multi-Mode Readers (BioTek, Winooski, VT, USA) at 510 nm.

### EdU assay

EdU assay was performed using a Cell-Light^TM^ EdU Apollo^®^567 in vitro Imaging Kit (RiboBio, Guangzhou, China) according to the manual instructions. 3T3-L1 cells cultured in GM were incorporated with 50 mM EdU for 2 h, followed by fixing with 4% paraformaldehyde. Then, the fixed cells were marked with Apollo reaction mixture, and the nuclei were stained with DAPI (Thermo Fisher Scientific). Cells were imaged via Nikon TE2000 microscope (Nikon).

### Chromatin immunoprecipitation

ChIP assays were performed using the Millipore ChIP Assay Kit (Millipore). Briefly, confluent 3T3-L1 cells were fixed with 1% formaldehyde for 5 min and then quenched, washed, collected, and lysed according to the manufacturer’s instructions of Simple ChIP Kit (Cell Signaling Technology, Danvers, Massachusetts, USA) to generate cross-link of protein-DNA complexes. Cell lysates were sonicated to generate chromatin fragments of 200–300 bp and immunoprecipitated using IgG as a negative control at 4 °C overnight. Precipitated chromatin DNA was collected and analyzed by ChIP-seq and ChIP-PCR. ChIP-seq reads were aligned to the mouse genome, and peaks were called with MACS software. The Great software was used for determining pathways significantly affected by HMGB2 binding genes. The MEME software was applied to analyze the sequences within HMGB2 peaks for enriched motifs. The primers of C/EBPβ for ChIP-PCR were showed in Supplementary Table 5.

### Dual-luciferase assay

The 2 kb DNA fragment of mouse C/EBPβ was synthesized by Sangon Biotech (Shanghai) Co., Ltd. Then, a series of truncated DNA fragments were inserted into Xho I and Hind III sites of pGL3-basic vector, together with pRL-TK (Promega) were cotransfected into HEK-293T cells, which were divided into two groups, one group is cotransfected with HMGB2-EGFP vector, and the other group is cotransfected with EGFP vector. After 48 h, cells were lysed for analysis of luciferase activities by using the dual-luciferase reporter assay system (Promega) according to the manufacturer’s instruction. The luciferase intensity was determined through dividing the relative fluorescence value for firefly luciferase by that of Renilla luciferase. Primers for a series of truncated DNA fragments were listed in Supplementary Table 6.

### Data analysis

All experiments were conducted at least three biological replicates, unless otherwise stated. All values are expressed as mean ± s.e.m., unless otherwise stated. qPCR data are normalized to GAPDH and expressed relative to WT (or control). *P < 0.05 and **P < 0.01 were taken as the criteria of statistical significance.

## Supplementary information

Supplementary Figure 1

Supplementary Figure 2

Supplementary Figure 3

Supplementary Figure 4

Supplementary Figure 5

Supplementary Figure 6

Supplementary Figure 7

Supplementary Figure Legends

Dataset 1

Dataset 2

Dataset 3

Dataset 4

Dataset 5

Dataset 6

Dataset 7

Dataset 8

Dataset 9

Dataset 10

Dataset 11

Supplementary Tables

## Data Availability

RNA-seq and ChIP-seq raw data have been uploaded in NCBI’s Sequence Read Archive (https://submit.ncbi.nlm.nih.gov/subs/sra/), BioProject ID: PRJNA694037.
