# Supplementary figures and images for: HMGB2 orchestrates mitotic clonal expansion by binding to the promoter of C/EBPβ to facilitate adipogenesis
Source: Cell Death Dis. 2021 Jul 2;12(7):666. doi: 10.1038/s41419-021-03959-3 (PMC8253743; doi:10.1038/s41419-021-03959-3)

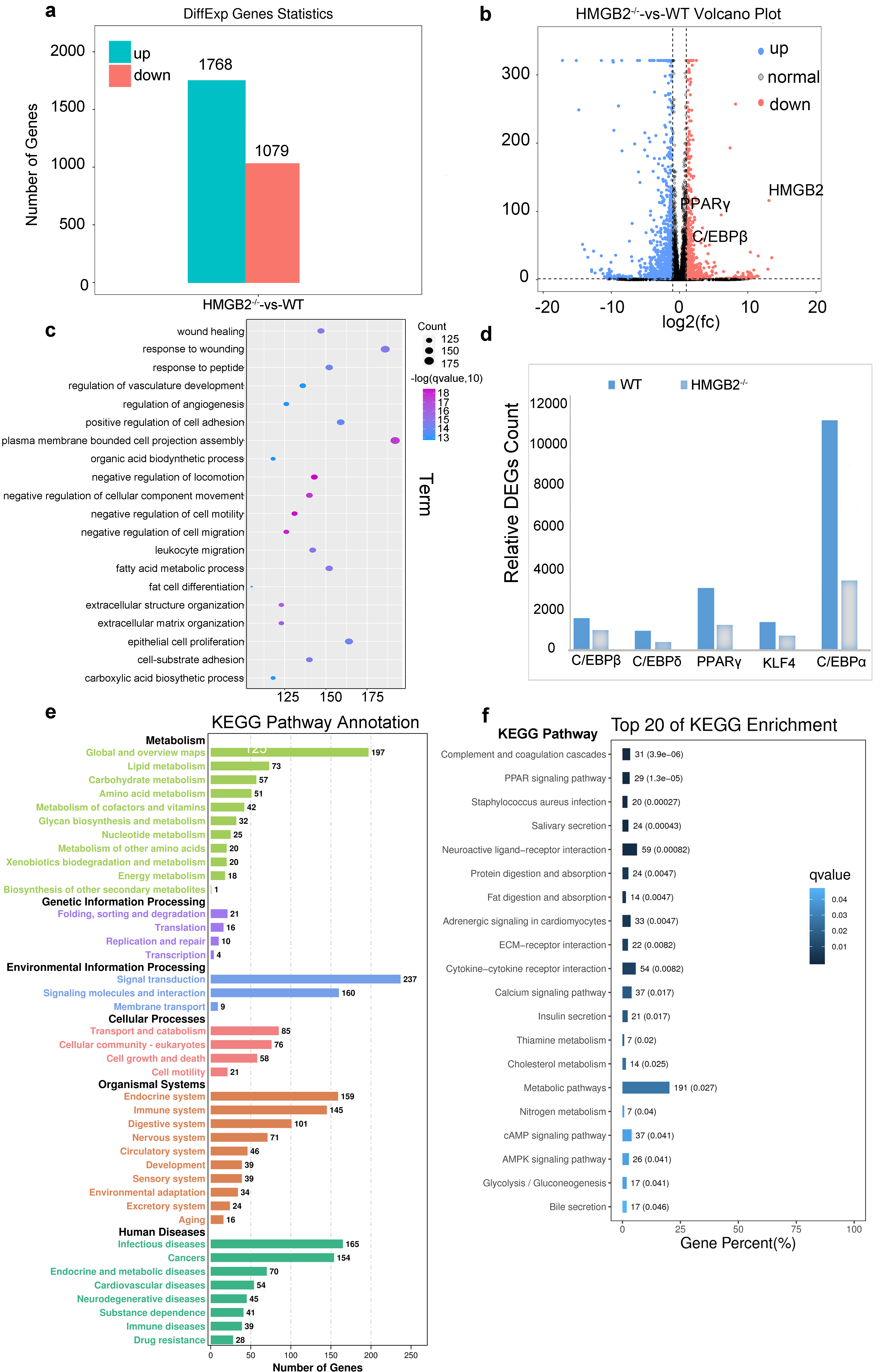

Supplement: Supplementary file 4 — Supplementary Figure 4 [file 41419_2021_3959_MOESM4_ESM.jpg]

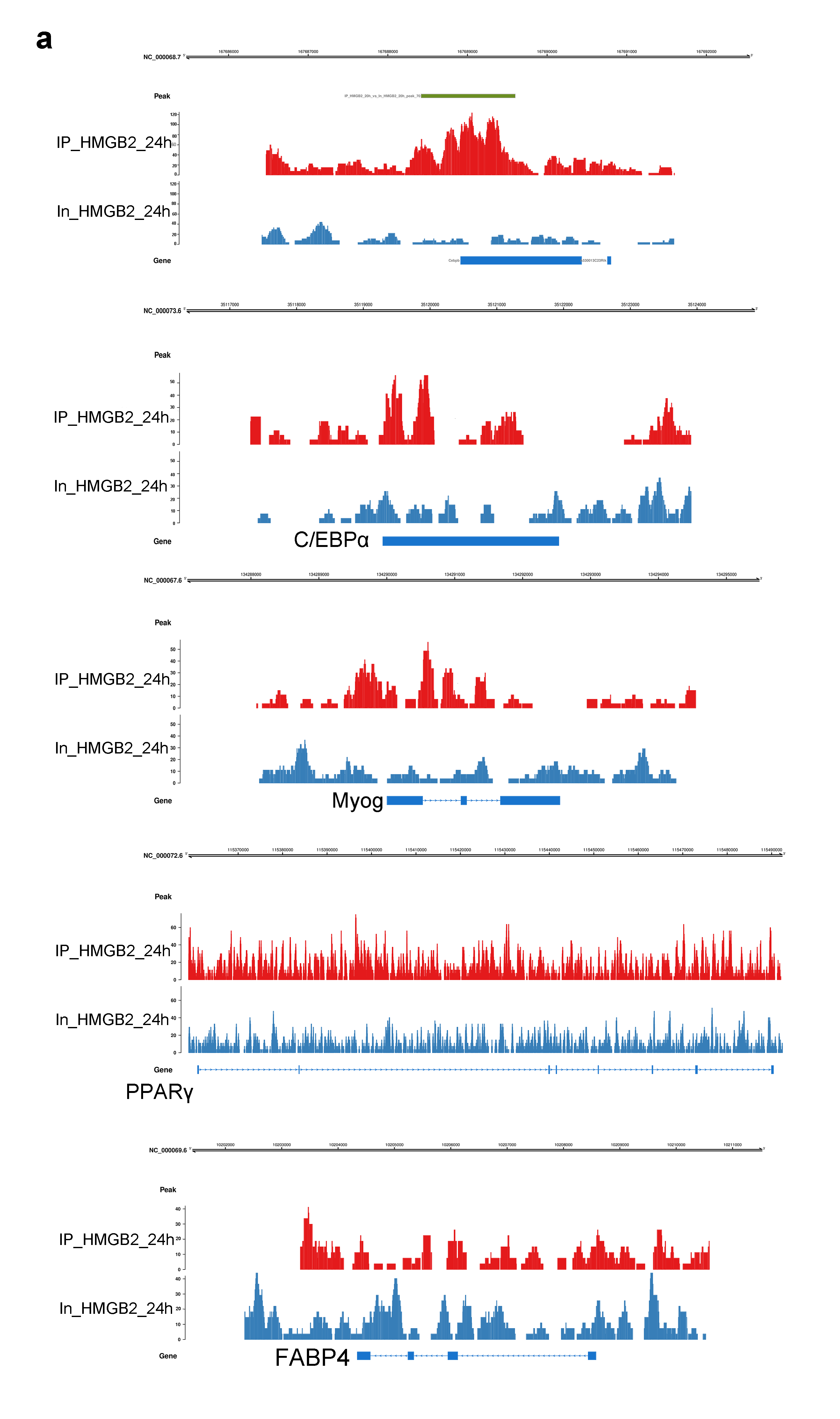

Supplement: Supplementary file 5 — Supplementary Figure 5 [file 41419_2021_3959_MOESM5_ESM.tif]

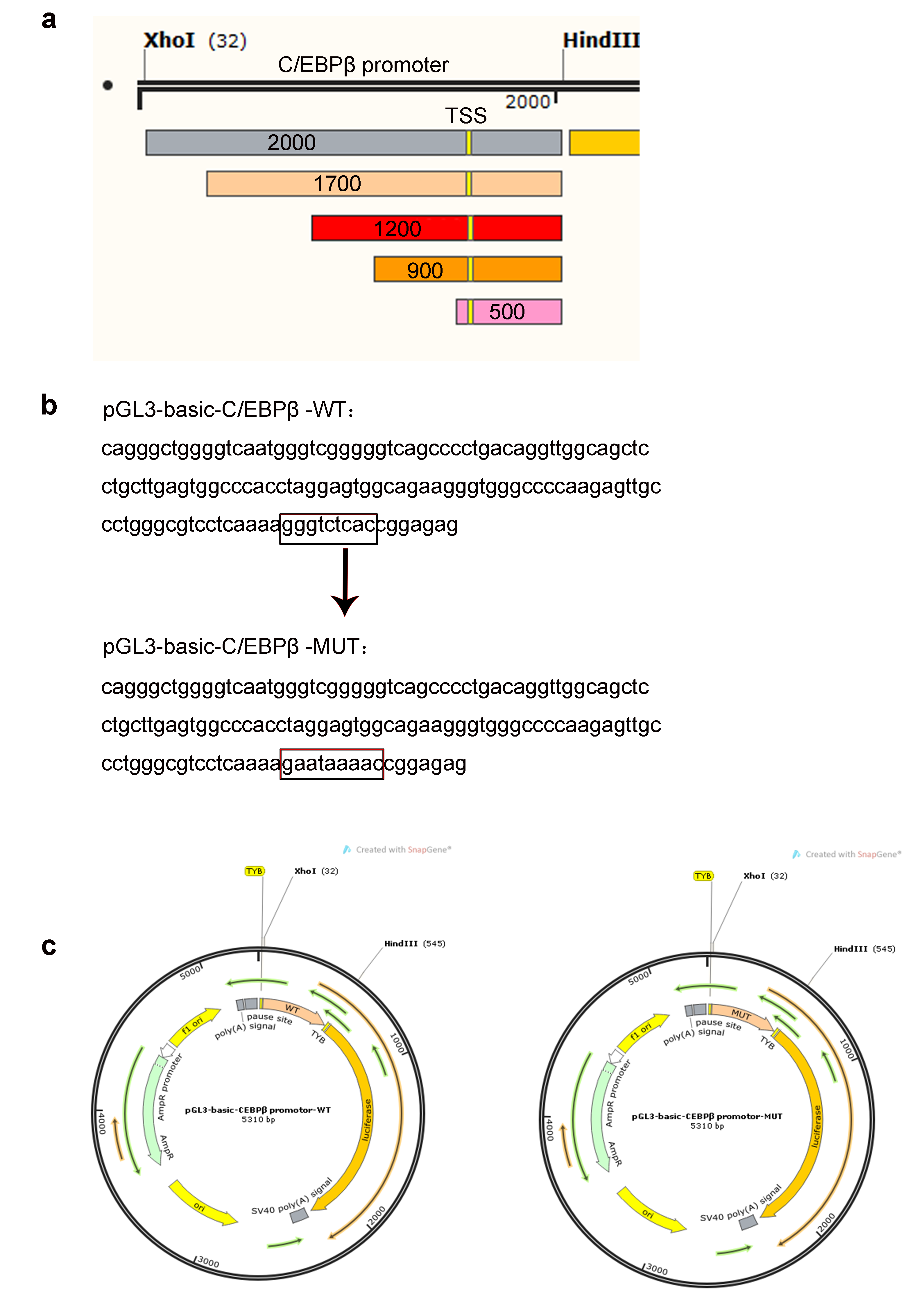

Supplement: Supplementary file 6 — Supplementary Figure 6 [file 41419_2021_3959_MOESM6_ESM.tif]

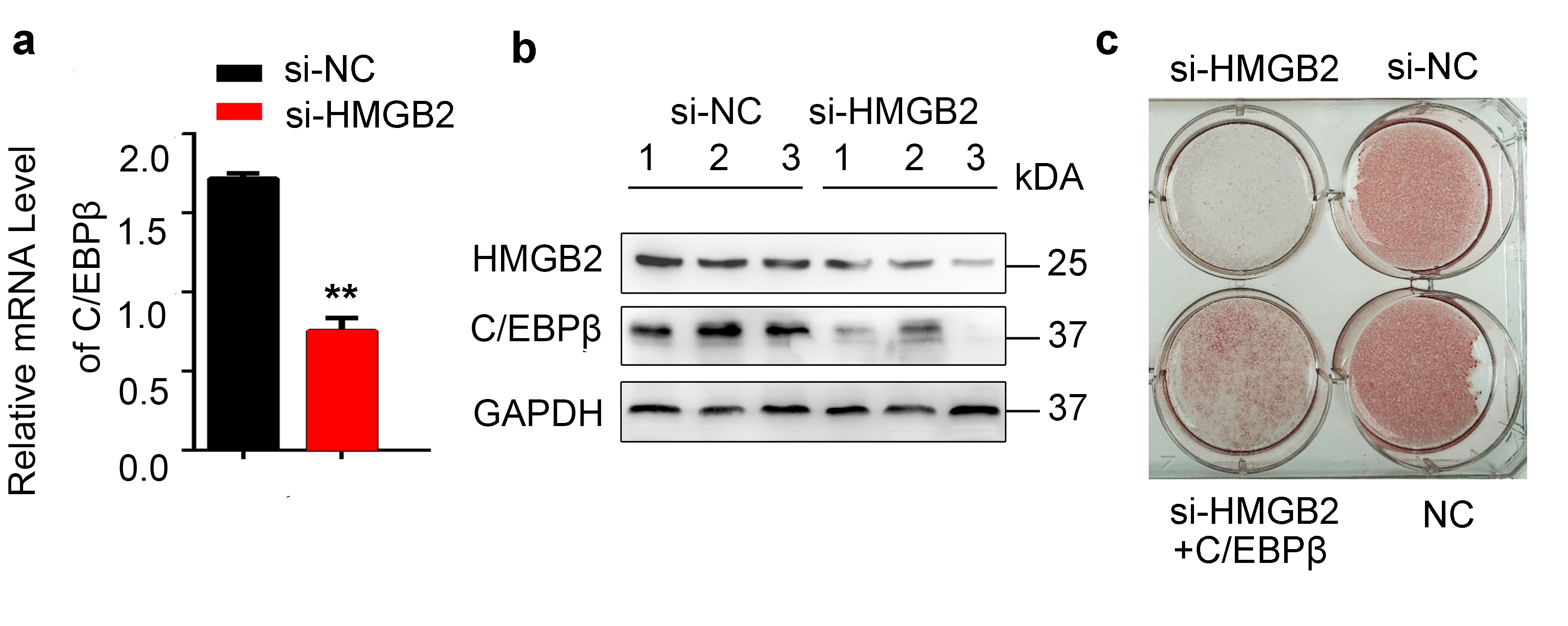

Supplement: Supplementary file 7 — Supplementary Figure 7 [file 41419_2021_3959_MOESM7_ESM.tif]
