# Supplementary material for: HMGB2 orchestrates mitotic clonal expansion by binding to the promoter of C/EBPβ to facilitate adipogenesis
Source: Cell Death Dis. 2021 Jul 2;12(7):666. doi: 10.1038/s41419-021-03959-3 (PMC8253743; doi:10.1038/s41419-021-03959-3)
Supplement: Supplementary file 8 — Supplementary Figure Legends [file 41419_2021_3959_MOESM8_ESM.doc]

**Supplementary Figure Legends**

**HMGB2 orchestrates mitotic clonal expansion by binding to the promoter of C/EBPβ to facilitate adipogenesis**

Keren Chen, Junyan Zhang, Feng Liang, Qi Zhu, Shufang Cai, Xian Tong, Zuyong He, Xiaohong Liu, Yaosheng Chen*, Delin Mo*

State Key Laboratory of Biocontrol, School of Life Sciences, Sun Yat-sen University, Guangzhou, Guangdong, China

* Corresponding author:

Yaosheng Chen, chyaosh@mail.sysu.edu.cn

Delin Mo, E-mail: modelin@mail.sysu.edu.cn，Fax: 020-39332940

**Supplementary Figure 1**. Design and confirmation of *hmgb2^-/-^* mice. **a** Schematic strategy of HMGB2 knockout *in vivo*. **b** PCR onfirmation of HMGB2 deletion in mice.

**Supplementary Figure 2**. Phenotype of *hmgb2*^-/-^ mice in contrast to WT. Four groups of mice: WT (chow diet), *hmgb2*^-/-^ (chow diet), WT-HFD and *hmgb2*^-/--^HFD. **a,** **b, c, d, e, f, g** Body weight, fat and lean weight, body size, ingWAT image, liver and H&E staining comparisons of four groups of mice. **h** Statistical data of abdominal fat cells size. (**a,** **b, c, d**: male mice, 20-week-old, n=5; Four groups: WT-chow diet, *hmgb2*^-/-^ mice-chow diet, WT with HFD and *hmgb2*^-/-^ mice with HFD)

**Supplementary Figure 3**. ChIP-seq analysis of HMGB2 enrichment at different stage in adipogenesis. **a** Sample correlation in -48h, 24h and 96h. The square of Pearson correlation coefficient (R2)＞0.92. **b** Reads distribution in 2kb upstream and downstream of TSS. Results was analyzed by deeptools (version: 2.5.4). **c** PCA Analysis of 2kb distribution in upstream and downstream of TSS. Principle component 1(PC1) and principle component 2 (PC2) are shown as scatter plots of x-axis and y-axis respectively. **d** KEGG enrichment in -48h and 96h in contrast to 24h.

**Supplementary Figure 4.** Effect of HMGB2 knockout on gene expression of ingWAT. **a** Statistical analysis for differential expression genes. The screening conditions are based on FDR<0.05 and |log2FC|>1, fold change>2. **b** Volcano plot for DEGs in *hmgb2*^-/-^ and WT mice. X axis: log values of difference multiples. Y axis: negative log10 values of different FDR. Red dot: up-regulated genes. Blue dot: down-regulated genes. Black dot: genes without change. **c** GO-BP clustering analysis of DEGs between *hmgb2*^-/-^ and WT mice. Data was processed by z-score, logFC＞0.5；P value＜0.05 and Q value ＜0.05. **d** Expression profile of key adipogenic genes in transcriptome. **e** KEGG pathway annotation for all DEGs. **f** Top 20 of KEGG pathways selected according to the q-value ranked lowest. Y axis: pathway. X axis: the number of genes as a percentage enriched.

**Supplementary Figure 5**. Enrichment of HMGB2 in C/EBPβ, C/EBPα, PPARγ and FABP4 at 24h.

**Supplementary Figure 6**. Construction for a series of C/EBPβ promoter fragments. **a** Schema of truncated DNA fragments of C/EBPβ promoter. **b** Mutation of HMGB2 binding site. **c** Snapgene profile of WT and mutation.

**Supplementary Figure 7**. Identification of relationship between HMGB2 and C/EBPβ. **a**, **b** Knockdown of HMGB2 led to decrease of C/EBPβ in 3T3-L1 cells. **c** Overexpressing C/EBPβ can partially rescue the inhibited adipogenesis caused by si-HMGB2, as confirm by Oil Red-O staining.
