# Supplementary material for: HMGB2 orchestrates mitotic clonal expansion by binding to the promoter of C/EBPβ to facilitate adipogenesis
Source: Cell Death Dis. 2021 Jul 2;12(7):666. doi: 10.1038/s41419-021-03959-3 (PMC8253743; doi:10.1038/s41419-021-03959-3)
Supplement: Supplementary file 20 — Supplementary Tables [file 41419_2021_3959_MOESM20_ESM.docx]

**Tables**

| Table S1 sgRNA for generating HMGB2*^-/-^* mice (5’ to 3’) | | |
| --- | --- | --- |
| Names | Sequence | PAM |
| 5'Guide | CGCGCCCCCGCGAGCGAGCG | CGG |
| 3'Guide | AGGTCTTAAGTCCTAGGTAG | AGG |

| Table S2 Primers for genotyping of HMGB2*^-/-^* mice (5’ to 3’) | | | |
| --- | --- | --- | --- |
| NO. | Name | Sequence | Product size |
| 1 | HMGB2-WT-F | GGTCCCAGAATGAACTGAGGGCTTC | WT: 387bp |
|  | HMGB2-WT-R | CCGCTGGGAATCTCAGGGAAAACTA |  |
| 2 | HMGB2-Mut-F | TCGCGGTGGCTTGTGTAAGTGTAAG | Mut: 431bp |
|  | HMGB2-WT-R | CCGCTGGGAATCTCAGGGAAAACTA |  |

| Table S3 The sequences of siRNAs targeting HMGB2 | |
| --- | --- |
| Name | Sequence |
| si-HMGB2-1 | F: CAAUGCUCCGAAGAGACCACCGUCU |
|  | R: AGACGGUGGUCUCUUCGGAGCAUUG |
| si-HMGB2-2 | F: GCCUGUCUAUUGGAGAUACUGCGAA |
|  | R: UUCGCAGUAUCUCCAAUAGACAGGC |
| si-HMGB2-3 | F: ACCGUAUGAGCAGAAAGCAGCUAAA |
|  | R: UUUAGCUGCUUUCUGCUCAUACGGU |

| Table S4 The primers for qPCR | |
| --- | --- |
| Name | Sequence |
| HMGB2 | F: AAGAGCGACAAAGCTCGTTATG |
|  | R: GCAGTATCTCCAATAGACAGGC |
| C/EBPβ | F: AAGCTGAGCGACGAGTACAAGA |
|  | R: GTCAGCTCCAGCACCTTGTG |
| PPARγ | F: GCATGGTGCCTTCGCTGA |
|  | R: TGGCATCTCTGTGTCAACCATG |
| FABP4 | F: ACAAGCTGGTGGTGGAATGTG |
|  | R: CCTTTGGCTCATGCCCTTT |
| C/EBPα | F: GGCTCTCATTCTTTTTGGTTTAGGG |
|  | R: CTAAGACCCACTACTACATACACCC |
| Fasn | F: CTGCGTGGCTATGATTATGGC |
|  | R: CTGCGTGGCTATGATTATGGC |
| LPL | F: TGCCACTTCAACCACAGCA |
|  | R: ACATTCCCGTTACCGTCCATC |
| Adipoq | F: CGATTGTCAGTGGATCTGACG |
|  | R: CAACAGTAGCATCCTGAGCCCT |
| Ccd25 | F: AAAAGCAGTTCTTGAAGATAG |
|  | R: AGAGCCTGGTACATATTGG |
| Cyclin D1 | F: CAGCAGGAGCTAAAGCCGAA |
|  | R: TCGTTGAGGAGGTTGGCATC |
| Cyclin E2 | F: ATGTCAAGACGCAGCCGTTTA |
|  | R: GCTGATTCCTCCAGACAGTACA |
| Mcm3 | F: TGGGAAGGACCACAACTC |
|  | R: CACTTTCTCCGCCCTAAT |
| Cdc45l | F: CGGAAGTGACGGAACACC |
|  | R: CAGTCGCTATCCTGTGAA |
| Gins1 | F: TGCCAGGGAGGAAACCAT |
|  | R: TAGGCTCAACCAATAGAAGTGC |
| CDK2 | F: GCTCTCCTTGCGTTCCATCC |
|  | R: ACGTGCCCTCTCCAATCTTC |
| CDK4 | F: ATGGCTGCCACTCGATATGAA |
|  | R: TCCTCCATTAGGAACTCTCACAC |
| P27 | F: CAAACTCTGAGGACCGGCAT |
|  | R: TCTTAATTCGGAGCTGTTTACGTC |
| Rb | F: TGCATCTTTATCGCAGCAGTT |
|  | R: GTTCACACGTCCGTTCTAATTTG |
| Cyclin A2 | F: AAGAGAATGTCAACCCCGAAAAA |
|  | R: ACCCGTCGAGTCTTGAGCTT |
| CD36 | F: GGTCCTTACACATACAGAGTTCGTTAT |
|  | R: CATTGGGCTGTACAAAAGACACA |
| Leptin | F: GAGACCCCTGTGTCGGTTC |
|  | R: CTGCGTGTGTGAAATGTCATTG |
| Glut4 | F: GTGACTGGAACACTGGTCCTA |
|  | R: CCAGCCACGTTGCATTGTAG |
| Hsl | F: CCAGCCTGAGGGCTTACTG |
|  | R: CTCCATTGACTGTGACATCTCG |
| Adrb3 | F: GGCCCTCTCTAGTTCCCAG |
|  | R: TAGCCATCAAACCTGTTGAGC |
| Irs1 | F: CGATGGCTTCTCAGACGTG |
|  | R: CAGCCCGCTTGTTGATGTTG |
| Irs2 | F: CTGCGTCCTCTCCCAAAGTG |
|  | R: GGGGTCATGGGCATGTAGC |
| Irs3 | F: TCGGCTCACCGTTTCCTTG |
|  | R: TCGCTCTCGTAGCACTCCA |
| Resistin | F: AAGAACCTTTCATTTCCCCTCCT |
|  | R: GTCCAGCAATTTAAGCCAATGTT |
| GAPDH | F: CATGGCCTTCCGTGTTCCTA |
|  | R: TGCCTGCTTCACCACCTTCT |

| Table S5 The primers of C/EBPβ for ChIP-PCR | |
| --- | --- |
| Name | Sequence |
| ChIP C/EBPβ-1 | F: GAGGGAACAGATAGTGTGCT |
|  | R: AGCCAGGCCTGGCTGTAAAGT |
| ChIP C/EBPβ-2 | F: CCACCTAGGAGTGGCAGAAG |
|  | R: GAGGCTTCTCAGGTGATTGC |
| ChIP C/EBPβ-3 | F: AACACTGTCCACGGAGGGAA |
|  | R: ACTCCTGGGAAGCAGAACTC |

| Table S6 The primers for truncated DNA fragments of C/EBPβ promoter | |
| --- | --- |
| Name | Sequence |
| P1-2000 | This DNA fragment was synthesized by Sangon Biotech Co., Ltd. |
| P2-1700 | F: GGGCTCGAGCAGGGCTGGGGTCAATGGGT |
|  | R: GCCAAGCTTTGGCGTCGGCGGGGCTCGGCGT |
| P3-1200 | F: GGGCTCGAGGACAGCGAGCATCCCCGGGGT |
|  | R: GCCAAGCTTTGGCGTCGGCGGGGCTCGGCGT |
| P4-900 | F: GGGCTCGAGCGCGCTCCTCCGTGTTCCCG |
|  | R: GCCAAGCTTTGGCGTCGGCGGGGCTCGGCGT |
| P5-500 | F: GGGCTCGAGGACCGGGACGCAGCGGAGC |
|  | R: GCCAAGCTTTGGCGTCGGCGGGGCTCGGCGT |
